# Purification of Sponges by Boiling

**Published:** 1901-03-09

**Authors:** 


### PURIFICATION OF SPONGES BY BOILING.

A boiled sponge is a spoiled sponge, as everyone knows, and thus while the disinfection of almost everything else used in an operation is effected by heat, the cleansing of sponges has to be done in other and not always entirely satisfactory ways. Indeed, so greatly do some surgeons doubt the trustworthiness of some of the methods in common use that, notwithstanding the manifold advantages of sponges, they prefer not to admit such dangerous material within the operation area. Dr. Elsberg,[1] however, some time ago made certain experiments which go far to show how the difficultties connected with the boiling of sponges may be got over. Sponge, he says, consists of an albuminoid material which is perfectly precipitated from its solution by a solution containing two per cent. of tannic acid and one per cent. of caustic potash. Hence, as it seemed to him, it should be perfectly possible to boil sponges in such a solution without spoiling them. In fact it might be said that the spongy material could not be dissolved in a solution from which it would be at once precipitated. On testing this theory in practice he found it to be correct. The solution could be used again and again, and after infected sponges had been treated in this way bacteriological investigation showed them to have been rendered absolutely sterile. The mode of procedure was as follows: The sponges were freed from calcareous matter by immersion for twenty-four hours in 8 per cent. muriatic-acid solution and then thoroughly washed in water. They were next boiled for fifteen minutes in a solution containing one part of potassium hydrate and two parts of tannic acid to 100 parts of water. The sponges were then washed and squeezed in sterile water, or in carbolic-acid or corrosive-sublimate solution until the potassium hydrate and tannic acid solution had been removed. They were then preserved in a 3 or 5 per cent. solution of carbolic acid.

[1] New York Med. Rec.

